# Transcriptome features of innate immune memory in *Drosophila*

**DOI:** 10.1371/journal.pgen.1010005

**Published:** 2022-10-17

**Authors:** Naoyuki Fuse, Chisaki Okamori, Ryoma Okaji, Chang Tang, Kikuko Hirai, Shoichiro Kurata

**Affiliations:** Graduate School of Pharmaceutical Sciences, Tohoku University, Sendai, Japan; Instituto Leloir, ARGENTINA

## Abstract

Immune memory is the ability of organisms to elicit potentiated immune responses at secondary infection. Current studies have revealed that similar to adaptive immunity, innate immunity exhibits memory characteristics (called "innate immune memory"). Although epigenetic reprogramming plays an important role in innate immune memory, the underlying mechanisms have not been elucidated, especially at the individual level. Here, we established experimental systems for detecting innate immune memory in *Drosophila melanogaster*. Training infection with low-pathogenic bacteria enhanced the survival rate of the flies at subsequent challenge infection with high-pathogenic bacteria. Among low-pathogenic bacteria, *Micrococcus luteus* (Ml) and *Salmonella typhimurium* (St) exerted apparent training effects in the fly but exhibited different mechanisms of action. Ml exerted training effects even after its clearance from flies, while live St persisted in the flies for a prolonged duration. RNA sequencing (RNA-Seq) analysis revealed that Ml training enhanced the expression of the immune-related genes under the challenge condition but not under the non-challenge condition. In contrast, St training upregulated the expression of the immune-related genes independent of challenge. These results suggest that training effects with Ml and St are due to memory and persistence of immune responses, respectively. Furthermore, we searched for the gene involved in immune memory, and identified a candidate gene, *Ada2b*, which encodes a component of the histone modification complex. The *Ada2b* mutant suppressed Ml training effects on survival and disrupted the expression of some genes under the training + challenge condition. These results suggest that the gene expression regulated by Ada2b may contribute to innate immune memory in *Drosophila*.

## Author summary

Innate immune memory is a memory-like character of the innate immune system and is one of the current hot topics, because of a great possibility for clinical applications. It is known that Bacillus Calmette Guérin (BCG) vaccine promotes the protection against not only tuberculosis but also a wide variety of pathogens in heterologous manners. Recent studies have demonstrated that innate immune memory is responsible for the vaccine effects of BCG. It has also been revealed that epigenetic reprogramming plays important roles in innate immune memory. However, the mechanisms underlying it are not fully understood yet. It is also unclear how much the mechanisms of innate immune memory are evolutionarily conserved among organisms.

Here we established an experimental system for detecting innate immune memory in *Drosophila*. We found that training infection of low-pathogenic bacteria enhanced the survival rate of flies after challenge infection of high-pathogenic bacteria. Especially, *Micrococcus luteus* (Ml) and *Salmonella typhimurium* (St) showed apparent training effects in fly. Our RNA-Seq analysis revealed that training effects of Ml and St could be attributed to memory and persistence of immune reactions, respectively. We also identified Ada2b, a component of the histone modification complex, as a factor involved in innate immune memory.

## Introduction

The immune machinery, which protects the host against pathogens, comprises innate immunity and adaptive immunity. Innate immunity is the primitive immune system that is evolutionarily conserved among multicellular organisms. As the innate immune system recognizes the molecular patterns of pathogens, the innate immune responses are not specific to a pathogen but function in a heterologous manner [1]. In contrast, adaptive immunity, which is evolutionarily developed in the lineage of vertebrates, specifically recognizes pathogens. Moreover, adaptive immunity exhibits the characteristics of memory, which mediates the potentiation of immune responses to secondary infections. Previously, innate immunity was not suggested to exhibit memory characteristics. However, recent studies have reported the memory characteristics of innate immunity, which are called "innate immune memory," "immune priming," "trained immunity," and "systemic acquired resistance" [2–5]. In this study, we use "innate immune memory" as a broad sense term.

Studies on invertebrates, which lack adaptive immunity, have reported the memory characteristics of innate immunity. For example, primary infection from the tapeworm parasite protects the copepod *Macrocyclops albidus* from secondary infection [6]. Similar phenomena have been observed in various species of invertebrates [2,3], and also in mammals [4]. The immune responses in severe combined immunodeficiency (SCID) mouse, which has a deficient adaptive immune system, can be potentiated at secondary infection [7], suggesting memory functions of the innate immune system. Bacillus Calmette Guérin (BCG) vaccine has been widely used for protecting infants against infections from *Mycobacterium tuberculosis*. The long history of BCG vaccine usage has revealed that this vaccine not only protects against tuberculosis but also against a wide variety of pathogens in a heterologous manner. Recent studies have demonstrated that the innate immune system mediates the heterologous effects of the BCG vaccine [8,9]. Thus, innate immune memory has attracted attention in the scientific community, and several researchers focus on elucidating the underlying molecular mechanisms.

The innate immune cells of mammals (such as macrophages and monocytes) respond to the pathogen-associated molecular patterns (PAMPs) and modulate their gene expression *in vitro* and *in vivo*. Previous studies have demonstrated that the responses of gene expression elicited after primary stimulus were enhanced or suppressed after secondary stimulus, suggesting "potentiation" or "tolerance" of immune responses, respectively [10–14]. For example, the expression of anti-microbial peptide (AMP) genes is potentiated during the secondary stimulus, whereas that of genes encoding pro-inflammatory mediators (such as interleukins) is suppressed. These modifications of gene expression are believed to represent innate immune memory at the molecular level. Furthermore, the epigenetic regulation of gene expression plays an important role in immune memory. Histone modifications, such as the methylation of histone H3 Lys4 (H3K4) and the acetylation of histone H3 Lys14 (H3K14) are dynamically modulated by immune stimuli, resulting in the modification of gene expression in response to secondary stimuli [11–14]. Epigenetic reprogramming is observed in short-lived peripheral myeloid cells (such as macrophages and monocytes) and long-lived stem cells (such as hematopoietic stem and progenitor cells: HSPCs) [15,16], consistent with the long-lasting effects of the innate immune memory in individuals. Moreover, metabolic shifts of cholesterol synthesis and glycolysis in innate immune cells are observed under immune training conditions and contribute to the potentiation of immune responses [17,18]. Thus, various data have been accumulated for innate immune memory although the underlying mechanisms have not been completely elucidated. For example, the mechanism involved in the conversion of immune information into epigenetic information during immune memory has not been elucidated. Additionally, the organization of immune responses at the individual level during primary and secondary infections is unclear.

We study the innate immune memory of *Drosophila melanogaster*. *Drosophila* is a suitable model in which various genetic tools can be employed for elucidating the molecular mechanisms at the individual level. In the last decade, analysis of innate immunity in *Drosophila* has revealed various molecular networks [1,19] and demonstrated that these molecules are well-conserved between insects and mammals. For example, the Toll and Imd pathways are two major signaling pathways of *Drosophila* immunity and correspond to Toll-like receptor (TLR) and tumor necrosis factor receptor (TNF-R) signaling pathways in mammals, respectively. The Toll pathway is activated mainly by gram-positive bacteria and fungi, while the Imd pathway is activated mainly by gram-negative bacteria. However, these responses are not exclusive and are overlapping to some extent [20–22]. These signaling pathways regulate the expression of immune-related genes, such as those encoding AMPs and peptidoglycan recognition proteins (PGRPs). In addition to this humoral response, the cellular response of immunity is mediated by fly hemocytes (corresponding to mammalian macrophages). Hemocytes phagocytose the pathogens and incorporate the killed pathogens into cellular vesicles. Furthermore, non-immune cells, such as the gut, muscle, neuronal, and reproductive organ cells respond to pathogen infection [23]. These responses are coordinated at the individual level and modulate physiology, metabolism, behavior, and consequently homeostasis of individual flies.

It is still not clear how much the mechanisms involved in innate immune memory are evolutionarily conserved among organisms. Previous studies have reported the phenomena related to immune memory in *Drosophila* [24–26]. In these experiments, the survival rates of flies subjected to primary infection increase after secondary infection. However, these training effects on survival rate can be attributed to immune persistence rather than immune memory [10,27]. Immune persistence involves the maintenance of primary infection-mediated immune activation during secondary infection. Meanwhile, in the case of immune memory, primary immune activation has ceased at the time of secondary infection but boosts immune responses against secondary infection. The mechanism involved in immune training in flies is unclear.

Here, we established experimental systems of immune training in *Drosophila*. RNA sequencing (RNA-Seq) analysis was performed to evaluate the memory and persistence of immune responses at the molecular level. A chromatin regulator potentially involved in innate immune memory was identified.

## Results

### Experimental system for *Drosophila* immune training

**Fig 1:**
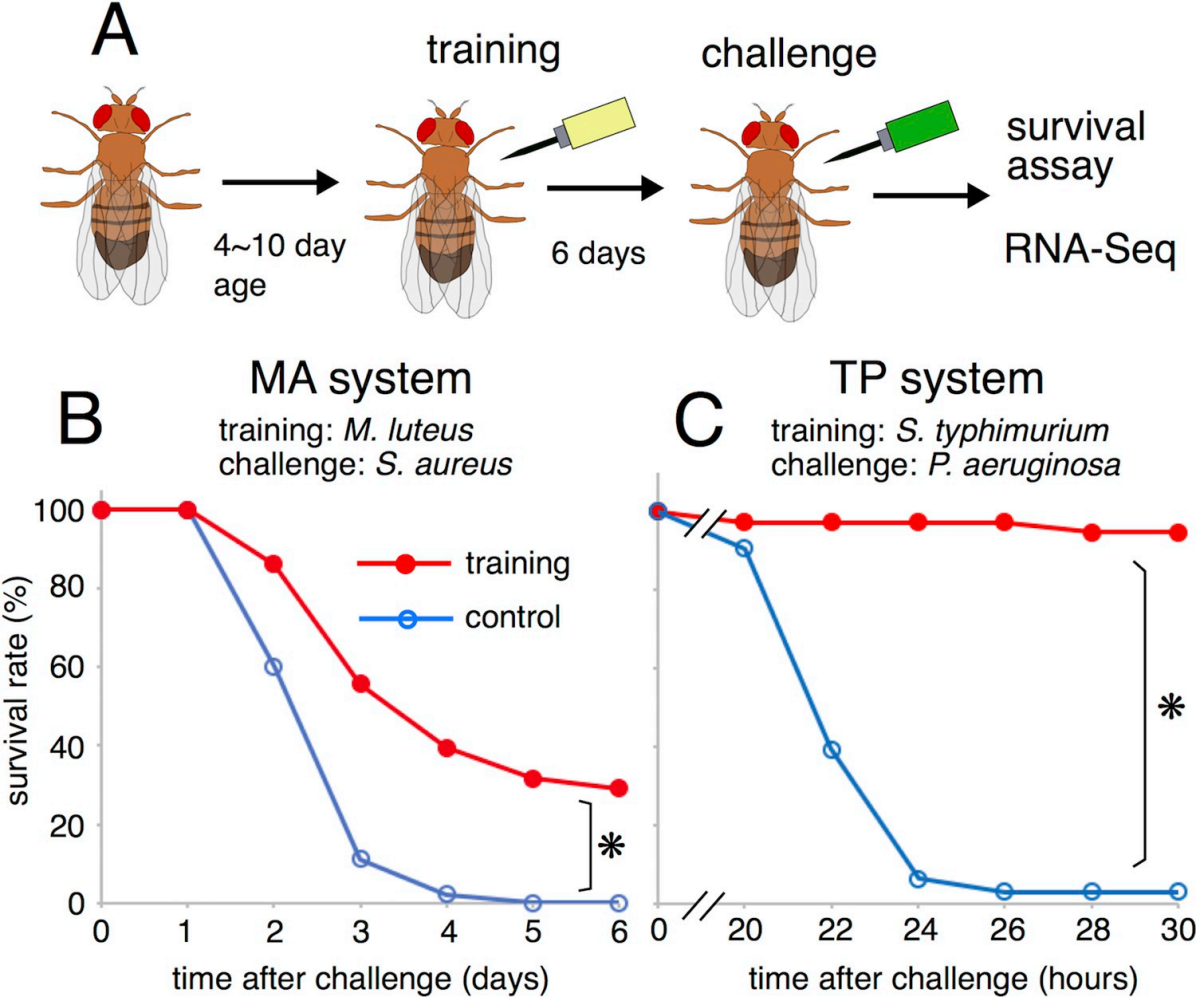
Experimental system of *Drosophila* immune training. (A) Schematic drawing of systemic infection experiment. After training and challenge injections, fly survival was monitored, and RNA-Seq analysis was performed. (B) Survival curves of MA system: Ml-training and Sa-challenge. Sa (OD = 0.5) was used for this experiment. (C) Survival curves of TP system: St-training and Pa-challenge. Red and blue lines represent the flies with and without training, respectively. Asterisks indicate statistically significant (p-value < 0.05) in Log-rank test. Numbers of flies used in these experiments are (B) 40, 57 flies / 3 vials, and (C) 42, 37 flies / 3 vials (with or without training, respectively).

We initially sought to establish an experimental system for detecting the immune training effects using *Drosophila*. Based on the effectiveness of live vaccines, the training effects of live bacteria were examined. Low-pathogenic bacteria were injected into wild-type flies (Oregon-R strain) for training (Fig 1A). On day 6 post-training, high-pathogenic bacteria were injected into the flies for challenge, and the survival rates of the flies were examined. The control flies were injected with saline for training. Various combinations of low-pathogenic bacteria (*Micrococcus luteus* (Ml), *Salmonella typhimurium* (St), *Staphylococcus saprophyticus* (Ss), and *Erwinia carotovora carotovora 15* (Ec); see Materials and methods) for training and high-pathogenic bacteria (*Staphylococcus aureus* (Sa) and *Pseudomonas aeruginosa* (Pa)) for challenge were examined. The survival rates of the trained, challenged flies were compared with those of control untrained, challenged flies. For example, Sa challenge killed most of the untrained flies within a week. However, prior training with Ml significantly increased the survival rates of the flies after challenge with Sa (Fig 1B). Furthermore, Pa was highly pathogenic and killed all flies within a day. However, most of the flies trained with St survived for several days after challenge with Pa (Fig 1C).

The training effect did not appear to be specific and exhibited cross-reactivity (S1 Fig). The Ml training induced increase in the survival rates of the flies after Pa challenge, as similar to that after Sa challenge. Moreover, St exerted similar training effects against Sa and Pa challenges. However, some combinations of bacteria exerted specific training effects. Ss exerted training effects against Sa but not against Pa. In contrast, Ec exerted training effects against Pa but not against Sa. We speculated that the specificity of these training effects may reflect the broad specificity of the innate immune signaling pathways.

To further characterize the immune training effects, this study will focus on the following two experimental systems: Ml training and Sa challenge (MA system); St training and Pa challenge (TP system) (Fig 1B–1C).

### Persistence and removal of training bacteria

**Fig 2:**
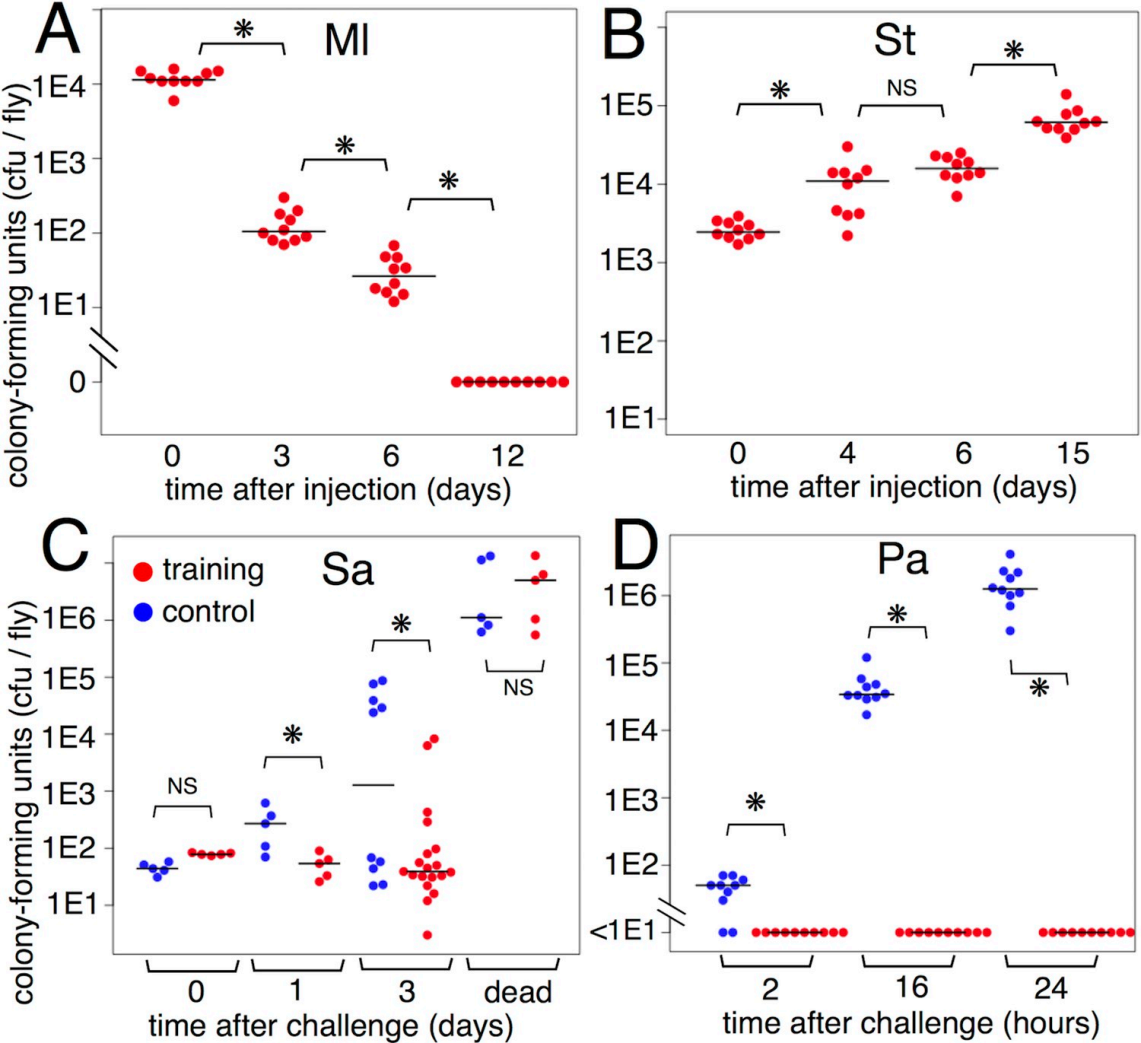
Bacterial load assay for training and challenge. (A, B) Loads of training bacteria, Ml (A) and St (B). The flies were collected at given times post-injection. (C, D) Loads of challenge bacteria, Sa-GFP (C) and Pa-Kan (D). The flies were collected at the indicated times post-challenge. "dead" means the flies died within 24 hours before sample preparation. Asterisks and NS indicate statistically significant (p-value < 0.05) and not significant, respectively, in Kruskal-Wallis ANOVA and post-hoc Wilcoxon rank sum test.

To characterize the training effects in the MA and TP systems, the persistence of training bacteria after injection was evaluated by measuring Ml and St load. Immediately after injection, the Ml load was approximately 12,000 colony-forming units (cfu) /fly, which gradually decreased each day thereafter (Fig 2A). On day 6 post-injection, only 0.5% of the initial load (31 cfu/fly) of Ml was detected. On day 12 post-injection, Ml was not detected in the fly. In contrast, the St load was high and increased to approximately 20-fold (relative to initial load) on day 15 post-injection (from 2,650 to 68,250 cfu/fly) (Fig 2B). Thus, Ml and St exhibit differential behaviors (Ml cells are removed, whereas St cells persist). A previous study [28] reported that St is incorporated into phagosomes of hemocytes and is viable for a prolonged duration. St was suggested to continuously activate the immune responses in fly.

Ml was completely absent in the fly on day 12 post-injection. At that time, the Ml training effect persisted and consequently increased the survival rates of flies after Sa challenge (S2A Fig), similar to the phenotypes on day 6 post-training. This indicates that live Ml cells are not needed to exert training effects at the time of challenge. However, the training effect of Ml did not appear to be permanent. Ml training did not increase fly survival after challenge on day 23 post-training (S2B Fig).

Next, the detrimental effects of immune training on fly longevity were examined (S3 Fig). The longevity of flies subjected to Ml training was similar to that of control flies. However, the longevity of flies subjected to St training was significantly lower (median survival time = 32 days) than that of the control (median survival time = 59 days), consistent with a previous study [28]. This result suggests that persistent St is advantageous for flies in short term as they enhance survival after subsequent infection but is detrimental for maintaining longevity in long term. In contrast, these negative effects were not detected in Ml training.

### Clearance of challenge bacteria and survival

Life or death after infection is the overall result of immunity (to remove pathogens), resistance (to protect host from pathogen-induced damage), and other physiological states [29]. To characterize the phenomena in the survival assay, the load of challenge bacteria was measured in live and dead flies. Antibiotic-resistant strains of challenge bacteria were used to distinguish them from training bacteria (see Materials and methods). In the TP system, the load of Pa markedly increased in the control flies but was completely absent within 2 h in the St-trained flies (Fig 2D). This all-or-none load is consistent with the survival rate in the TP system. Most of the control flies died after Pa challenge, whereas most of the St-trained flies survived (Fig 1C). Thus, St training stimulated the immunity of flies to clear Pa.

In the MA system, the load of Sa slowly increased in the live control flies. On day 3 post-challenge, two discrete populations of the live flies that had a high or a low load of Sa were observed (Fig 2C). The high Sa load in live flies was similar to that in dead flies on day 3. This suggests that the individual flies with a high Sa load may die shortly, while those with a low Sa load may survive for a prolonged period. After Ml training, the load of Sa was low in many flies, suggesting that Ml training potentiates fly immunity to suppress the growth of Sa. Moreover, the Ml-trained flies on day 3 exhibited a relatively broad distribution of Sa load. The Sa load in dead Ml-trained flies was high and similar to that in the dead untrained flies. These results suggest that Ml-trained flies may be resistant to middle ranges of Sa load.

### RNA-Seq analysis of immune training

**Fig 3:**
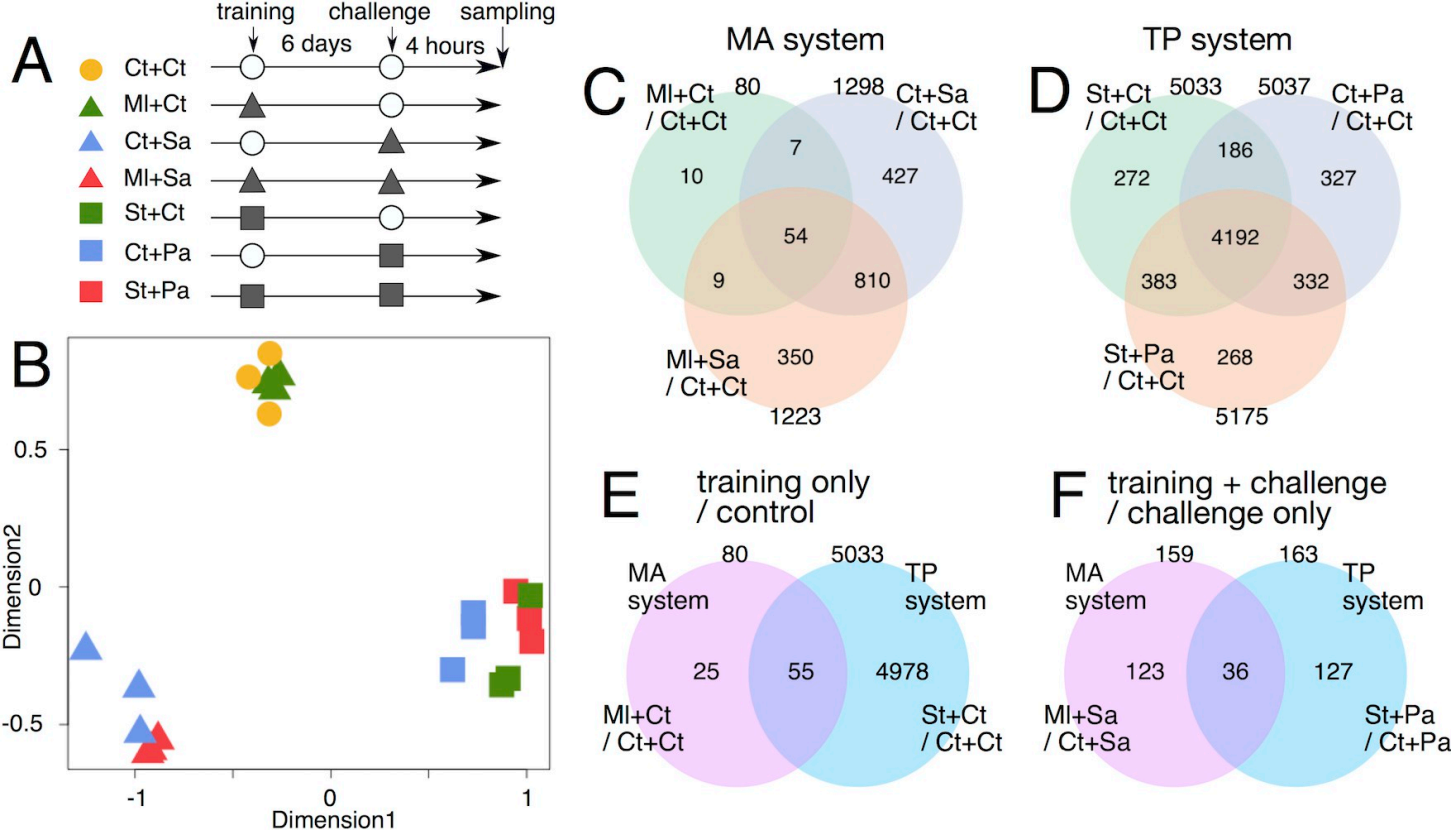
Overall features of transcriptomes for immune training. (A) Schematic drawing of the experimental schedule. Empty circles and filled symbols represent saline- and bacteria-injection, respectively. (B) MDS analysis of transcriptomes for all samples. Distance between plots correlates to the similarity of transcriptomes between samples. (C, D) Venn diagrams for DEGs detected from pair-wise comparisons against the control condition in MA system (C) and in TP system (D). (E, F) Venn diagrams for DEGs detected for training only against the control condition (E) and for training + challenge condition against challenge only condition (F) in MA and TP systems. The gene IDs of DEGs are listed in S3 Table.

RNA-Seq analysis was performed to elucidate the molecular mechanism of training effects. The experimental schedule for sample preparation is shown in Fig 3A. Wild-type adult flies (Oregon-R) were injected with training bacteria (Ml or St) or saline (designated as Ct), followed by the injection of challenge bacteria (Sa or Pa) or saline (Ct) at day 6 post-training. The flies were collected at 4 h post-challenge. Thus, the fly samples were prepared under the following seven conditions in the MA and TP systems: control (Ct + Ct), Ml training only (Ml + Ct), Sa challenge only (Ct + Sa), Ml training + Sa challenge (Ml + Sa), St training only (St + Ct), Pa challenge only (Ct + Pa), and St training + Pa challenge (St + Pa) conditions. Three biological replicates (samples) were prepared for each condition (21 samples in total). Total RNA was extracted from the whole body of flies and subjected to sequencing analysis. The sequence reads were mapped to the *Drosophila* reference genome (S1 Table). The read counts of each gene were normalized between all samples (S1 Data for MA system and S2 Data for TP system).

To obtain an overview of the transcriptome profiles, Multidimensional scaling (MDS) analysis was performed (Fig 3B). In the MA system, the transcriptome profile of the training only (Ml + Ct: green triangles) was similar to that of the control (Ct + Ct: yellow circles). Meanwhile, the transcriptome profile of the challenge only (Ct + Sa: blue triangles) was similar to that of the training + challenge (Ml + Sa: red triangles). In the TP system, the transcriptome profile of the training only (St + Ct: green squares) was distinct from that of the control (Ct + Ct: yellow circles) but was similar to that of the challenge only (Ct + Pa: blue squares) and training + challenge (St + Pa: red squares). Thus, the transcriptome of the Ml training is close to the steady state, while that of the St training was similar to the state of immune activation. This is consistent with the result of bacterial load analysis (clearance of Ml and persistence of St in flies).

Pairwise comparisons between the conditions identified differentially expressed genes (DEGs) (S2 and S3 Tables). In the MA system, the number of DEGs between the control (Ct + Ct) and training only (Ml + Ct) (80 genes in total, 11 genes in fold-change ≥ 2) was lower than that between the control (Ct + Ct) and challenge only (Ct + Sa) (1298 genes in total, 357 genes with a fold-change ≥ 2). The DEGs under three conditions (training only, challenge only, and training + challenge conditions against control condition) were compared (Fig 3C). The training only and challenge only shared 61 DEGs, while the challenge only and training + challenge shared 864 DEGs, suggesting that the state of Ml training is different from that of Sa challenge.

In the TP system, the number of DEGs between the control (Ct + Ct) and training only (St + Ct) (5033 genes in total; 1377 genes with fold-change ≥ 2) was similar to that between the control (Ct + Ct) and challenge only (Ct + Pa) (5037 genes; 1337 genes in fold-change ≥ 2) (S2 and S3 Tables). The DEGs under the three conditions were compared against the control condition in the TP system (Fig 3D). The training only and challenge only shared 4378 DEGs. Additionally, the challenge only and training + challenge shared 4524 DEGs, suggesting that the state of St training is similar to that of Pa challenge.

Next, the DEGs in the MA and TP systems were compared, which revealed 55 common DEGs for the training only condition against control condition (Fig 3E). These genes will be responsive to training in both MA and TP systems. Gene Ontology (GO) analysis revealed that the common DEGs were enriched to the genes related to GO:0045087-innate immune response (11-fold enrichment, p-value = 3.4E−5). Interestingly, the common DEGs comprised two members of the Turandot family (TotA and TotX) and five members of the Bomanin family (BomS5, BomT1, BomT2, BomBc2, and BomBc3). Moreover, other members of these families were included in the DEGs specific to Ml and St training. The DEGs specific to Ml training comprised BomS6 and its regulator (Bbd), while those specific to St training comprised TotC, TotM, BomS1, BomS2, BomS3, BomS4, BomBc1, and BomT3. The results suggest that these secreted proteins might contribute to the training effects in the MA and TP systems.

Additionally, 36 genes were identified as the common DEGs between MA and TP systems for training + challenge conditions against challenge only conditions (Fig 3F). These genes will be responsive to training effects under challenge conditions in both MA and TP systems. Again, the common DEGs were enriched to the genes related to GO:0045087-innate immune response (26-fold enrichment, p-value = 1.4E−7). However, these genes did not contain Turandot or Bomanin family members but comprised several AMPs (e.g. Drosomycin, Metchnikowin, Attacin-A, and Cecropin A1). This suggests that enhanced expression of some AMPs under training + challenge conditions may be common in MA and TP systems.

### Gene expression patterns under training and challenge

**Fig 4:**
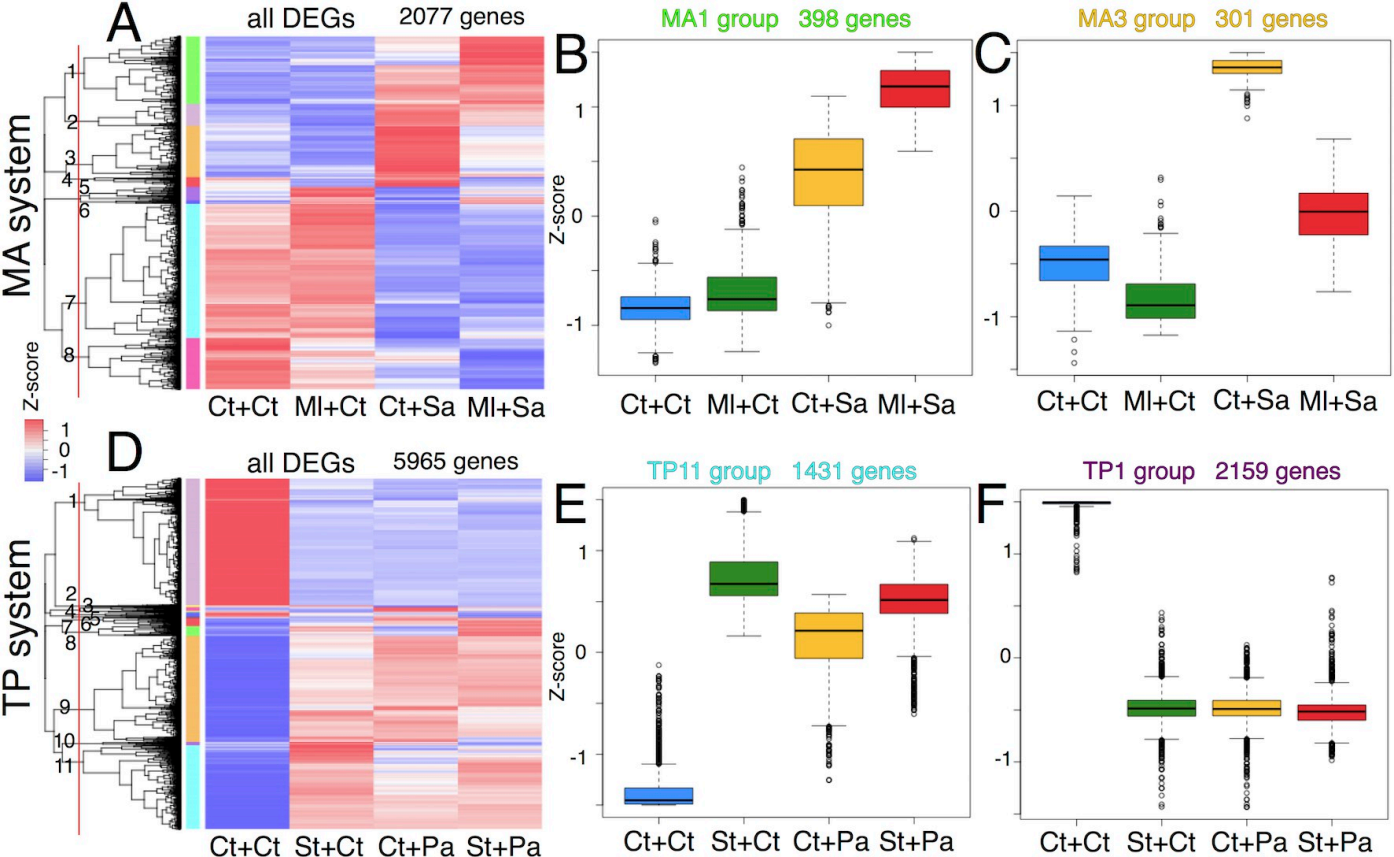
Clustering analysis of DEGs. (A, D) Heat maps of z-scores for DEGs of MA (A) and TP (D) systems. Genes (rows) were ordered by clustering analysis. Red lines represent thresholds on dendrograms to divide the DEGs into the clustering groups (numbers and color bars). (B, C, E, F) Box plots of z-scores of DEGs categorized to MA1 (B), MA3 (C), TP11 (E), and TP1 (F) groups. The numbers of genes categorized to these groups are indicated in graphs.

To characterize the patterns of gene expression under training and challenge conditions, the Z-scores (normalized deviations of expression) were calculated for total DEGs (2077 and 5965 genes in the MA and TP systems, respectively). The heatmap of Z-scores clustered according to genes (in rows) was displayed (Fig 4). The overall view of heatmaps clearly revealed that the transcriptome of the Ml training only was similar to that of the control but was different from that of the challenge only and training + challenge in the MA system (Fig 4A). In contrast, the transcriptome of the St training only was different from that of the control but was similar to that of the challenge only and training + challenge in the TP system (Fig 4D). These ovarall features indicate that Ml training returned to the steady state, whereas St training maintained the immune-active state at the time of challenge. These transcriptome data together with the results of survival and bacterial load assays (Figs 2 and 3) suggest that Ml and St exert training effects through immune memory and persistence, respectively.

To further characterize DEGs, the DEGs were categorized into 8 and 11 clustering groups for the MA and TP systems, respectively (Fig 4A and 4D; colored bars), and the expression pattern of each clustering group was analyzed. In the MA system, 398 genes of the MA1 group were upregulated under challenge conditions and were further stimulated under training + challenge conditions (Fig 4B). Meanwhile, 301 genes of the MA3 group were upregulated under challenge conditions but were suppressed under training + challenge conditions (Fig 4C). Thus, the expression patterns of the MA1 and MA3 groups recaptured "potentiation" and "tolerance" of gene expression in innate immune memory, respectively (see Introduction). In the TP system, most DEGs were categorized into the TP11 group (upregulated by any stimulus) (Fig 4E) or the TP1 group (downregulated by any stimulus) (Fig 4F). Other clustering groups in the MA and TP systems exhibited diverse expression patterns (S4 and S5 Figs). Among them, some groups of the MA and TP systems exhibited similar expression patterns. For example, the genes of the MA5, MA6, and TP8 groups were upregulated by training but not by challenge (S4 and S5 Figs).

**Fig 5:**
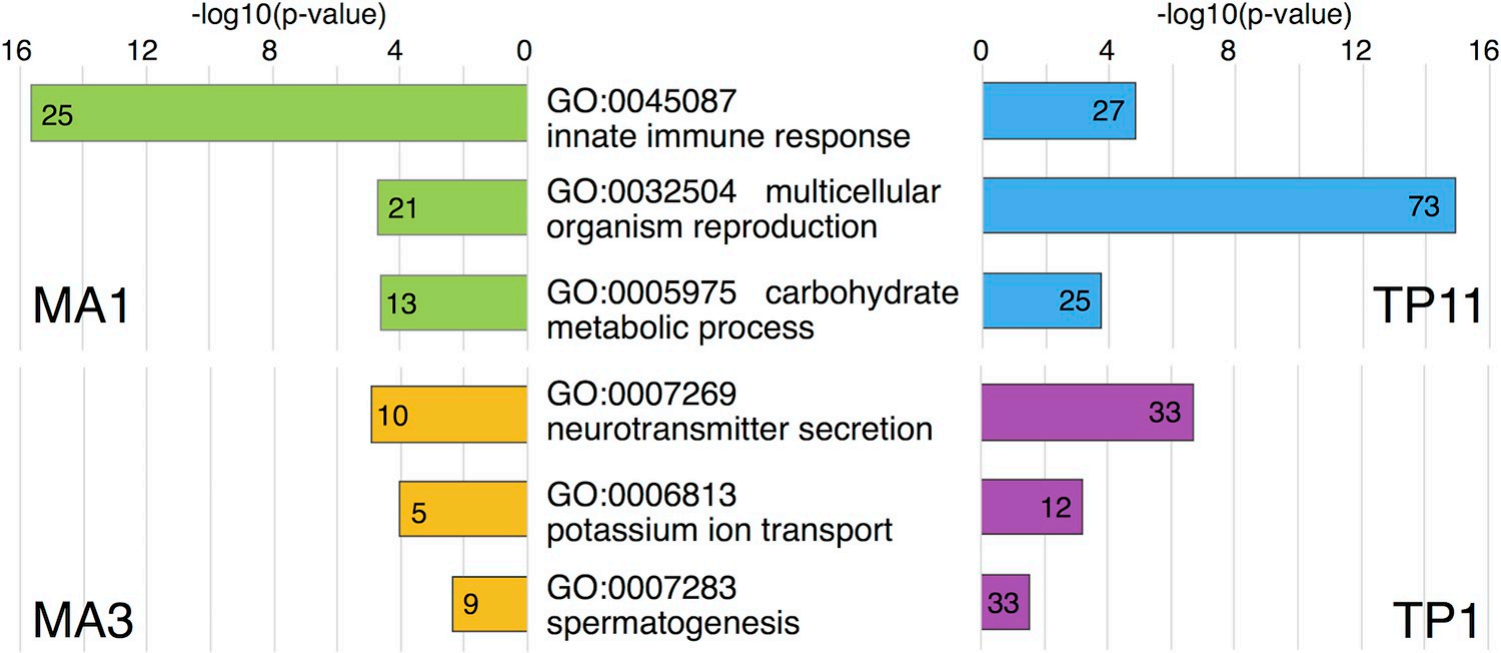
GO analysis for the clustering groups. GO analysis (GOTERM_BP_DIRECT category) for each clustering group was performed (S4 and S5 Tables). MA1 (green bars) and TP11 (blue bars) groups shared some enriched GO terms. Among them, three representative GO terms are shown here. Bar graphs show p-values of each GO term, as -log10 values. Numbers under the top of bars indicate numbers of genes matched to the GO terms. MA3 (yellow bars) and TP11 (magenta bars) groups also shared some enriched GO terms.

GO analysis was performed for each clustering group. The immune-related genes were significantly enriched in some of the clustering groups. In particular, the MA1 group of the MA system and the TP11 group of the TP system were enriched in "GO:0045087-innate immune response" (Fig 5). The MA1 group shared 124 genes with the TP11 group, and the shared genes included several AMPs (*Drosomycin*, *Attacin-A*, *Attacin-B*, *Cecropin C*, *Cecropin B*, *Cecropin A2*, and *Cecropin A1*). For example, expression patterns of some genes (*Drosomycin*, *Cecropin A1*, and *Attacin-A*) were shown in S6 Fig. The immune-related GO terms were also detected for other clustering groups, MA5 and TP8 (S4 and S5 Tables). However, the gene expression of these groups were different from those of the MA1 and TP11 groups and were upregulated under training conditions (S4 and S5 Figs). The MA5 and TP8 groups comprised some members of the Turandot and Bomanin families (*TotA*, *TotX*, *TotC*, and *BomS6* genes for the MA5 group; *BomT2* and *BomT1* genes for the TP8 group). Thus, the immune-related genes will be regulated in several different modes during immune training.

To observe typical expression patterns of the immune-related genes under the training and challenge conditions, the gene lists of published data were utilized. A previous study analyzed gene expression in *Drosophila* after infection with 10 bacterial species and identified the infection-responsive core genes that are commonly upregulated or downregulated in response to most of the 10 bacteria [22]. This gene list included AMPs, PGRPs, Turandot, Bomanin genes, and other classical targets of the Toll and Imd pathways. We used this gene list to analyze the expression of the infection-responsive core genes during immune training. Heatmaps of fold-change of gene expression (S7A and S7B Fig) revealed that the expression patterns of several core upregulated genes were similar to those of the MA1 group in the MA system and the TP11 group in the TP system. This suggests that the MA1 group in the MA system and the TP11 group in the TP system represent typical expression patterns for the infection-responsive core upregulated genes. The expression patterns of core downregulated genes were similar to those of the MA7 group in the MA system (S7C and S4E Figs; repression by Sa challenge) but were not typical in the TP system (S7D Fig). This suggests that most of the core downregulated genes may be independent of immune training in MA and TP systems. As a control, the expression of housekeeping genes was analyzed, and did not show typical patterns under training and challenge conditions (S7E and S7F Fig).

To explore the biological functions of DEGs, GO terms other than the immune-related functions were examined. The MA1 and TP11 groups were enriched with the genes related to "GO:0032504-multicellular organism reproduction" and "GO:0005975-carbohydrate metabolic process" in addition to "innate immune response” (Fig 5). The MA3 and TP1 groups were enriched with genes related to "GO:0007269-neurotransmitter secretion," "GO:0006813-potassium ion transport," and "GO:0007283-spermatogenesis" (Fig 5). MA4, MA8, and TP1 groups were enriched with the genes related to "GO:0010906-regulation of glucose metabolic process" (S8 and S9 Figs). MA8, TP7, and TP9 groups were enriched with the genes related to "GO:0006120-mitochondrial electron transport, NADH to ubiquinone." MA6 and TP10 groups were enriched with the genes related to "GO:0030431-sleep." Other GO terms enriched to the clustering groups are listed in S4 and S5 Tables. These biological functions listed from GO analysis may be related to immune training in MA and TP systems. To evaluate this possibility, future studies must examine whether the genes related to these biological functions contribute to training effects.

### Involvement of Ada2b in innate immune memory

The results of this study suggest that the MA system represents an example of immune memory in *Drosophila*. The expression pattern of the MA1 group will recapture the potentiation of immune responses during innate immune memory at the molecular level. Therefore, this study focused on the mechanism underlying the expression of the MA1 genes. The database of publications was searched for enrichment of the genes of the MA1 group, and one study on Ada2b, a component of the histone modification complex, [30] was identified (on FlyMine analysis, 52 matches, p-value = 1.0E−44). This previous study performed microarray analysis of *Ada2b* mutant and identified the Ada2b-regulated genes that comprised several immune-related genes. We used the list of the Ada2b-regulated immune-related genes (see Materials and methods, 62 genes [30]) to analyze their expression patterns in our RNA-Seq data. We found that many of the genes exhibited the MA1-like expression pattern (boosted activation by training + challenge) (S10A and S10C Fig). In contrast, the Ada2b-independent immune-related genes (30 genes [30]) rarely exhibited this expression pattern (S10B and S10D Fig). Thus, the genes enriched in the MA1 group are not only immune-related genes but also Ada2b-regulated genes. Based on these observations, we hypothesized that Ada2b might be involved in immune memory.

**Fig 6:**
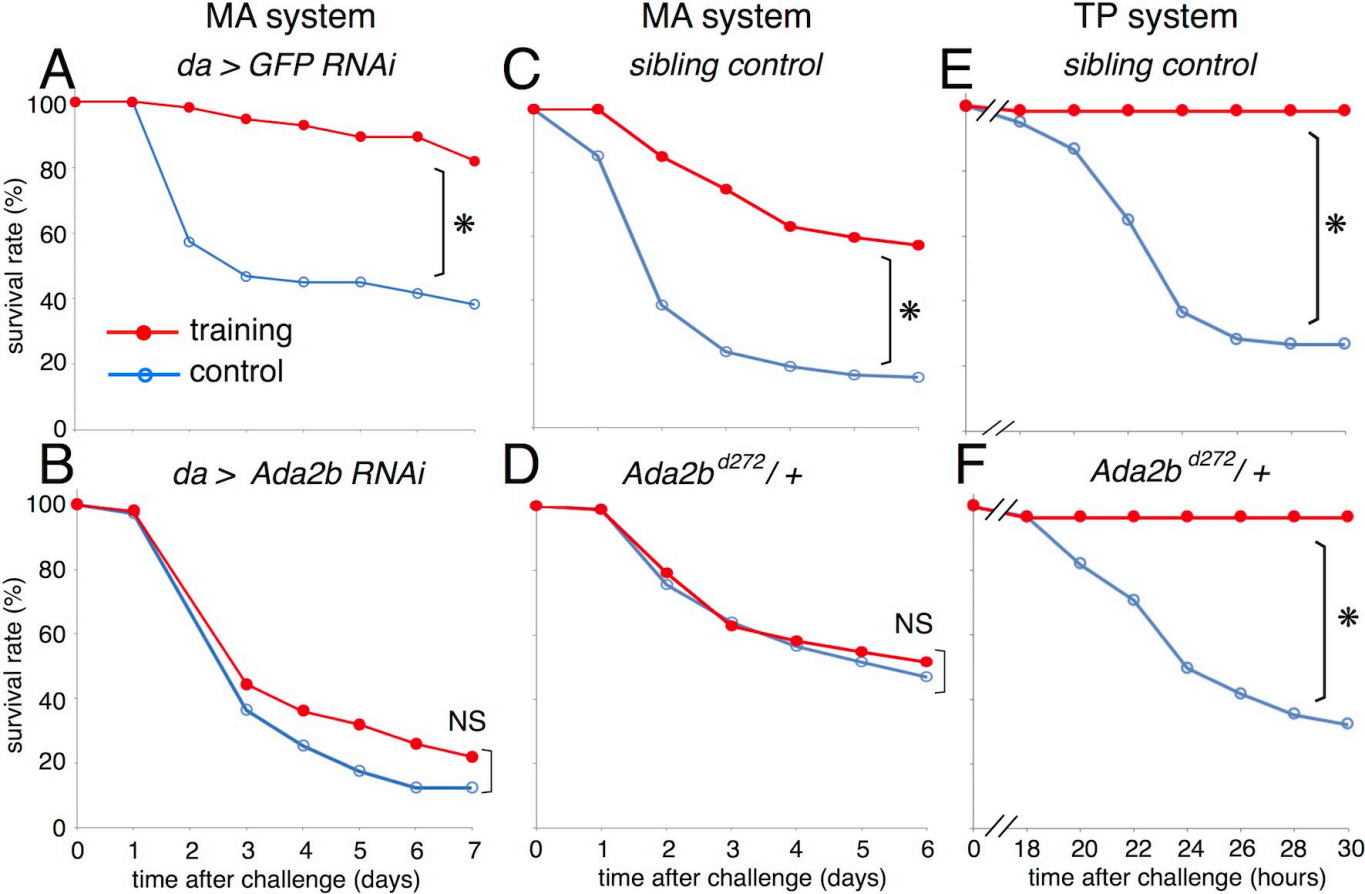
Involvement of Ada2b in Ml-training effects. (A-D) Survival curves of flies under Ml-training and Sa-challenge. (A) Control line (da > GFP RNAi), (B) *Ada2b* knock down line (da > *Ada2b* RNAi), (C) Sibling control line (*w*; *TM6* / +), (D) *Ada2b* heterozygotes (*w*; *Ada2b* ^d272^ / +). (E, F) Survival curves of flies under St-training and Pa-challenge. (E) Sibling control line (*w*; *TM6* / +), (F) *Ada2b* heterozygotes (*w*; *Ada2b* ^d272^ / +). Pa (OD = 1E-2) was used in these experiments, because these fly lines showed strong resistance against Pa when compared with Oregon-R (Fig 1C). Red and blue lines represent the flies with and without training, respectively. Asterisks and NS indicate statistically significant (p-value < 0.05) and not significant, respectively, in Log-rank test. Numbers of flies used in these experiments are (A) 56, 57 flies / 3 vials, (B) 96, 100 flies / 6 vials, (C) 80, 92 flies / 6 vials, (D) 97, 103 flies / 6 vials, (E) 52, 60 flies / 3 vials, (F) 58, 62 flies / 3 vials (with or without training, respectively).

Firstly, we examined whether expression of *Ada2b* itself is changed under training and challenge conditions. RNA-Seq data showed nearly constant expression of *Ada2b* under training and challenge conditions in the wild-type flies (S11B Fig). Next, the effects of *Ada2b* knockdown were examined using the *Ada2b RNAi* line. Reverse transcription polymerase chain reaction (RT-PCR) analysis revealed that the ubiquitous expression of *Ada2b RNAi* (*da-Gal4*, *UAS-Ada2b RNAi* line) knocked down the expression of *Ada2b* mRNA to approximately half of that of the control level (S11A Fig). Then, the survival assay in MA system was performed in the *Ada2b RNAi* line. Ml training did not increase the survival rate of the *Ada2b RNAi* flies after Sa challenge, while the control line (*da-Gal4*, *UAS-GFP RNAi*) exhibited apparent training effects of Ml (Fig 6A and 6B). Moreover, the phenotypes of the *Ada2b* mutant heterozygotes (*Ada2b* ^d272^/+) [31] were examined as *Ada2b* homozygotes are lethal during development. The effect of Ml training was abolished in *Ada2b* heterozygotes but retained in the sibling control (Fig 6C and 6D). These results indicate that Ada2b is required for the survival enhancement mediated by Ml training in the MA system. Furthermore, the training effect of St in the TP system was examined. The *Ada2b* heterozygotes and the sibling controls both exhibited increased survival rates after Pa challenge under the St training condition (Fig 6E and 6F). These results indicate that the *Ada2b* heterozygotes retained St training effects and also physiological immune responses, but lost the Ml training effects.

We next addressed which tissue Ada2b functions in. As hemocyte is one of the major immune cells in *Drosophila*, the hemocyte-specific knockdown experiment was carried out using the *hmlΔ-Gal4* line. We found that knockdown of *Ada2b* in hemocytes (*hmlΔ -Gal4*, *UAS-Ada2b RNAi*) diminished training effects of Ml, while the control line (*hmlΔ-Gal4*, *UAS-GFP RNAi*) exhibited apparent training effects of Ml (S12 Fig). These results suggest that Ada2b might function in hemocytes for innate immune memory.

**Fig 7:**
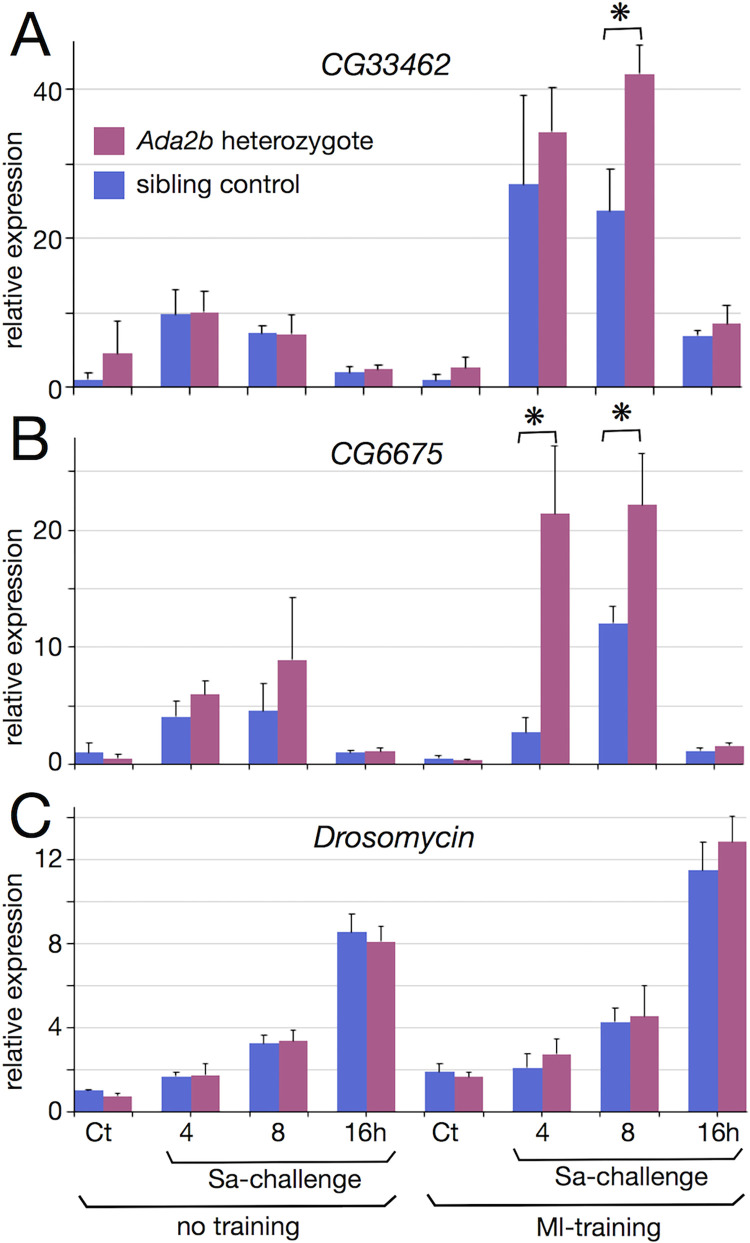
Involvement of Ada2b in gene expression under Ml training. (A-C) RT-PCR analysis measured relative expression of the *CG33462* (A), *CG6675* (B), and *Drosomycin* (C) genes (against the *RpL32* control gene). Sibling control line (*w*; *TM6* / +) (blue bars) and *Ada2b* heterozygotes (*w*; *Ada2b* ^d272^ / +) (magenta bars) were injected with Ml or saline, were re-injected with Sa, and were collected at 4, 8, and 16 h post-challenge. Ct means the control with non-challenge. Asterisks indicate statistically significant (p-value < 0.05) differences between the sibling control and the *Ada2b* heterozygotes judged by one-way ANOVA and Tukey HSD post-hoc tests. No marks indicate not significant differences between fly lines.

To examine the roles of Ada2b in immune memory, gene expression in the *Ada2b* heterozygotes was examined. The fly samples (the *Ada2b* mutant heterozygote and the sibling control) with or without Ml training were collected at 4, 8, and 16 h post-Sa challenge and subjected to RT-PCR analysis. The expression levels of *CG33462*, *CG6675*, and *Drosomycin* genes (which belong to MA1 group and encode a putative peptidase, a putative lipase, and an AMP, respectively) were examined (S6A, S11C and S11D Figs). In the control line, the expression of *CG33462* was induced by Sa challenge and was further potentiated by Ml training + Sa challenge (Fig 7A). Surprisingly, the expression of *CG33462* in the *Ada2b* heterozygotes was upregulated when compared with that in the sibling control at 8 h post-challenge. Similarly, the expression of *CG6675* in the *Ada2b* heterozygotes was upregulated when compared with that in the sibling control at 4 and 8 h post-challenge (Fig 7B). These differential expressions between the *Ada2b* heterozygote and the control line were detected only under Ml training conditions but not under no training conditions. These results suggest that Ada2b is involved in the repression of gene expression under the training + challenge condition and that gene regulation via Ada2b may ensure appropriate levels of gene expression to exert training effects. Meanwhile, the *Drosomycin* expression in *Ada2b* heterozygotes was similar to that in the sibling control (Fig 7C), suggesting that the mutant phenotype might be specific to some of the genes. However, in the case of *Drosomycin*, the manifestation of training effects was slow (at 16 h post-challenge) when compared with that analyzed using RNA-Seq data (at 4 h post-challenge; S6A Fig). These phenomena can be attributed to different genetic backgrounds of the fly lines (the Oregon-R line for RNA-Seq versus the *w* mutant line for RT-PCR). Thus, some of the gene regulations during immune memory might be varied under genetic backgrounds.

Our results suggest that fine-tuning of gene expression via Ada2b may contribute to innate immune memory in *Drosophila*. As Ada2b is thought to be a component of the histone modification complex, it might be involved in epigenetic gene regulation during immune memory. However, the *Ada2b* heterozygotes increased the expression of some genes under the training + challenge condition (Fig 7A and 7B), suggesting that the potentiation of gene expression by Ml-training might involve the factor(s) other than Ada2b. Alternatively, it might be possible that our analysis using the *Ada2b* heterozygotes could not reveal entire functions of Ada2b. Further analyses are needed to clarify the roles of Ada2b and other factors in the gene expression under innate immune memory.

## Discussion

This study established the MA and TP experimental systems for detecting immune training effects in *Drosophila*. In the TP system, St persisted in flies for at least 15 days post-injection. The overall transcriptome of the St training was similar to the state of immune activation, suggesting that the training effect of St can be attributed to immune persistence. In contrast, the training effect of the MA system may be associated with immune memory because Ml exerted training effects even after its clearance from flies. Moreover, the transcriptome of the Ml training was similar to the state of control, but the expression of the immune-related genes was potentiated by Ml training after challenge. Thus, this study provides models of immune memory and persistence on training effects in *Drosophila*.

In the MA system, the training effects of Ml were retained at least for 12 days but were suppressed after 23 days. In the mammalian system, the epigenetic information is memorized in the HSPCs, and immune memory lasts as long as the HSPCs produce peripheral myeloid cells [15,16]. In *Drosophila*, it is not clear how long the stem (progenitor) cells produce hemocytes during adult stage although hemocytes gradually decline in number after 1 week post-eclosion [32–34]. As our study utilized the survival assay to evaluate immune memory, hidden memory at the molecular level may remain for a prolonged duration. A recent study demonstrated the transgenerational inheritance of innate immune memory in mammals [35], which has also been reported in invertebrates [3,36]. Future studies must examine if the innate immune memory in *Drosophila* is inherited by the next generation.

It is not clear which cells work for immune memory in *Drosophila*. Previous studies have suggested that tissue injury and apoptotic corpse stimulate hemocytes to potentiate immune responses against subsequent infection [37–39]. St-trained and Ml-trained hemocytes may share some of the characteristics described in these studies. Although hemocytes and fatbody are the main tissues for cellular and humoral immunity, non-immune cells, such as gut, neuronal, and reproductive cells, also respond to pathogen infection [23]. It might be possible that the interactions between tissues may organize immune memory at individual levels. To address this issue, the tissues and the genes involved in innate immune memory must be identified.

Our RNA-Seq analysis was performed using RNAs extracted from the whole body. Hence, the cells in which gene expression is modulated during immune memory are unknown. A recent single-cell RNA-Seq analysis revealed the transcriptome atlas of adult fly at the single-cell resolution and identified several new cell species, including subtypes of hemocytes and their progenitor cells [40]. This database would be valuable for addressing the tissue-specific gene expression for innate immune memory in the future.

In mammalian innate immune cells, immune training is reported to modulate gene expression representatively via two modes [11–14]. The upregulation of gene expression under challenge is enhanced or suppressed by prior training. The genes exhibiting potentiation and tolerance during immune memory are enriched for the AMP-encoding genes and the pro-inflammatory mediator-encoding genes, respectively. Thus, these studies in mammals suggest that immune training enhances the immunity to eliminate pathogens and suppresses the inflammatory response to prevent detrimental effects of immunity with both contributing to maintaining the homeostasis of the organism during innate immune memory.

In the *Drosophila* MA system, the expression pattern of the MA1 group corresponds to the potentiation mode under immune training conditions. Moreover, these genes were enriched in the immune-related genes, including several AMP-encoding genes. Thus, the training-mediated upregulation of immune-related genes is conserved between flies and mammals. The expression pattern of the MA3 group corresponds to tolerance mode under training conditions. However, these genes were not enriched for any GO term related to immunity but enriched for "neurotransmitter secretion." Thus, we speculated that the neurotransmitter molecules might contribute to immune memory at the individual level although further experiments are needed to evaluate this possibility. Previous studies have demonstrated that neural networks respond to and regulate immunity [35,41]. The role of neural networks in innate immune memory must be examined in future studies.

We exmianed the factors involved in innate immune memory of *Drosophila*, and identified a candidate, Ada2b. The survival enhancement resulting from Ml training was abolished in *Ada2b* RNAi and mutant lines. Moreover, the hemocyte-specific knockdown of *Ada2b* diminished training effects of Ml. These results suggest that Ada2b might function in hemocytes for innate immune memory. The expression of three genes belonging to the MA1 group was examined in the *Ada2b* mutant heterozygotes. Of these, the expression of two genes (*CG6675* and *CG33462*) was perturbed in the *Ada2b* heterozygotes under the training + challenge condition. This suggests that Ada2b may be involved in gene expression during immune memory. In contrast, *Drosomycin* expression was not changed in the *Ada2b* heterozygotes, suggesting that Ada2b might function specifically for some of the MA1 group genes.

Ada2b interacts with the HAT (histone acetyltransferase) module containing GCN5 and Ada3. The HAT module further associates with several other proteins to form large protein complexes (such as the SAGA complex) [30,31,42]. The Ada2b-containing complex is reported to be involved in the epigenetic regulation of gene expression. Previous studies have demonstrated that Ada2b is required for the acetylation of histone H3 Lys9 (H3K9) and H3 Lys14 (H3K14) *in vivo* [30,43]. Histone acetylation is generally considered to contribute to the activation of gene expression. Therefore, the upregulation of *CG6675* and *CG33462* in the *Ada2b* heterozygotes relative to the sibling control was surprising. However, a previous study demonstrated that some genes are downregulated in the *Ada2b* mutant, whereas other genes (including the immune-related genes) are upregulated in the mutant [30]. Therefore, we suggest that Ada2b may regulate gene expression positively and negatively in a complex manner although the possibility of indirect effects on gene expression cannot be ruled out. Alternatively, the functions of Ada2b can be potentially underestimated as our analysis was performed using the *Ada2b* heterozygotes. Importantly, gene expression was altered in the *Ada2b* heterozygote under the training + challenge condition but not under the training only or challenge only condition. Thus, the effect of the *Ada2b* mutant may be restricted to the condition of immune memory. Based on these findings, we hypothesized that the Ada2b-mediated epigenetic gene regulation contributes to innate immune memory.

We also consider the possibility that the potentiation of gene expression under Ml-training might involve the epigenetic regulators other than Ada2b. In mammalian system, it is known that some of the histone modifications, such as methylation of H3K4 and acetylation of H3K14, play important roles for innate immune memory [11–14]. In *Drosophila*, the histone modifications during immune memory are still unknown. Further studies are needed to clarify histone modifications and those regulators during *Drosophila* innate immune memory. Additionally, the evolutionary conservation of innate immune memory has not been evaluated yet. The *Drosophila* system established in this study will be useful to address these issues in the future.

## Materials and methods

### Flies

The following *Drosophila melanogaster* lines were used in this study: Oregon-R (Bloomington *Drosophila* Stock Center (BDSC) #6362), *da-Gal4* (BDSC#55851), *UAS-GFP RNAi* (BDSC#9330), *UAS-Ada2b RNAi* (National Institute of Genetics, Japan (NIG) #9638R-3), *white* ^1118^ (*w*) mutant (BDSC#3605), *hmlΔ-Gal4 UAS-2XEGFP* (BDSC#30140), and *Ada2b* ^d272^ mutant (a gift from Dr. N. Zsindely, University of Szeged) [24,25] lines. To knock down *Ada2b* ubiquitously, the *da-Gal4* line was crossed with the *UAS-Ada2b RNAi* line. The resulting progenies (*da-Gal4*, *UAS-Ada2b RNAi*; designated as *da > Ada2b RNAi*) were used for the experiment. The progenies resulting from the cross of *da-Gal4* and *UAS-GFP RNAi* lines were used as controls (*da-Gal4*, *UAS-GFP RNAi*; designated as *da > GFP RNAi*). To knock down *Ada2b* in hemocytes, the *hmlΔ-Gal4* line was used in a similar way. The *Ada2b* mutant heterozygotes (*Ada2b* ^d272^/+) were obtained by crossing the *w* line with the *w*; *Ada2b* ^d272^/*TM6* line. The siblings (*TM6*/+) of this cross were used as controls. Flies were reared on standard corn meal medium at 25°C.

### Bacteria

The following bacteria were used in this study: *Micrococcus luteus* (Ml: IFO:13276), *Salmonella typhimurium* (St: SL1344), *Staphylococcus saprophyticus* (Ss: GTC:0205), *Erwinia carotovora carotovora 15-GFP* (Ec) [44], *Staphylococcus aureus* (Sa: ATCC10801), *Pseudomonas aeruginosa* (Pa: ATCC15692), Sa-GFP (RN4220/pTetON-GFPopt, spectinomycin-resistant strain) [45], and Pa-Kan (PA01/pBBR1MCS2, kanamycin-resistant strain) [46]. Ml, St, Ec, Sa, and Pa were cultured in Luria-Bertani (LB) medium (Nacalai Tesque), while Ss was cultured in nutrient broth medium (Becton Dickinson). Sa-GFP and Pa-Kan were cultured in a medium supplemented with spectinomycin (100 μg/mL) and kanamycin (200 μg/mL), respectively. The culturing temperature for Ml was 30°C, while that for other bacteria was 37°C.

Bacteria used for training stage were cultured overnight (approximately 16 h). Next, the bacterial suspension was centrifuged at 2,000 x g for 10 min, and the bacterial pellet was resuspended in saline (Otsuka Pharmaceutical). The cell density of the suspension was adjusted to the required concentrations (optical density (OD) = 1, unless otherwise stated). For challenge stage, the overnight bacterial culture was diluted 100-fold and re-cultured for 3 h. Challenge bacteria were pelleted and diluted to the required concentrations (Pa and Pa-Kan, OD = 1E−5; Sa, OD = 0.2; Sa-GFP, OD = 0.1, unless otherwise stated) in saline.

### Survival assay

Infection experiments were performed as described previously [47]. Briefly, adult male flies aged 4–7 days were used for the experiments. The bacterial suspension or saline (control) (70 nL) was injected into the body cavity of the fly thorax using a micromanipulator (Drummond, Nanoject II) and a glass needle (Drummond, 3 1/2 inch capillary). Challenge injection was performed on day 6 post-training injection unless otherwise stated. The flies that died within 3 h of challenge were censored from the survival assay. After next day, the number of dead flies was counted every 2 h for Pa challenge or every day for Sa challenge. The survival curves were statistically compared using the Log-rank test, followed by Tukey’s honest significant difference (HSD) post-hoc test with the R program.

### Bacterial load assay

To measure the bacterial load, the flies were collected at the indicated time points post-injection and washed briefly with 70% ethanol. Next, one fly was placed into a tube and homogenized using a pestle in 100 μL of LB medium. The fly extract was serially diluted, and 10 μL of the diluted extract was spotted on LB agar plates. The plates were incubated at 30°C (Ml) or 37°C (St, Sa, and Pa) overnight. The bacterial load in the original fly extract was calculated and represented as colony-forming units (cfu) per fly (cfu/fly). In this protocol, no bacterial colonies developed after plating the extracts of control flies without training or challenge. To measure the load of challenge bacteria, antibiotic-resistant strains of challenge bacteria (Sa-GFP and Pa-Kan) were used. Fly extracts were spotted on LB agar plates containing antibiotics as described above. The bacterial loads were statistically compared using Kruskal-Wallis analysis of variance (ANOVA), followed by Wilcoxon rank sum post-hoc test with the R program.

### Sample preparation for RNA-Seq

The training and challenge infections of Oregon-R adult male flies were performed as described in the Results section. To analyze early and strong responses of gene expression against challenge infection, the challenge bacteria were injected at high concentrations (OD = 1 (Sa) or 1E−2 (Pa)) into flies. The flies were collected at 4 h post-challenge, rapidly frozen in liquid nitrogen, and stored at −80°C until RNA extraction. Total RNA was isolated from 10 pooled flies of each sample using TRIzol reagent (Thermo Fisher Scientific), following the manufacturer’s instructions. The yield and purity of RNAs were evaluated using Nano-Drop (Thermo Fisher Scientific), Qubit (Invitrogen), and Bioanalyzer (Agilent). The sequencing libraries were prepared using a strand-specific RNA library prep kit (Agilent). Illumina HiSeq 2000 was used to perform sequencing (36 bases, single-end). The raw sequencing data were deposited in DNA Data Bank of Japan, Sequencing Read Archive (DDBJ DRA) (accession number: DRA008187).

### RNA-Seq data analysis

Transcriptome analyses were performed using the Linux or the Macintosh operating system. Computations were partially performed on the NIG supercomputer at the ROIS National Institute of Genetics. The adaptor sequences (Illumina TruSeq Adaptors) were removed from sequence data (fastq files) using Cutadapt [48] with the setting -O 20 -e 0.1 -m 50. The trimmed read sequences were mapped to the *Drosophila melanogaster* reference genome (ver. 6.04) using the Hisat2 program [49] with default parameters. Gene annotation data (ver. BDGP6.79) were utilized to attribute reads to genes. The number of reads was counted for each gene using Htseq [50] with the setting -s no -a 0 -m intersection-nonempty.

The read count data were analyzed using the R environment and initially normalized using the calcNormFactors function with the trimmed mean of M values (TMM) normalization method of the edgeR package [50]. Multidimensional scaling (MDS) analysis was performed using the plotMDS function of edgeR. Differentially expressed genes (DEGs) were identified using the glmLRT function of edgeR with a statistical criterion (generalized linear model likelihood ratio test, false discovery rate (FDR)-adjusted p-value < 0.05). Venn diagrams of DEGs were drawn using the venn.diagram function of the VennDiagram package. Z-scores of DEGs were calculated using the rowScales function of the scrime package. Clustering analysis of DEGs was performed using the heatmap function of the gplots package. Statistical analysis of gene expression was performed using ANOVA, followed by Tukey’s HSD post-hoc test.

The web-based databases DAVID [51], FlyMine [52], and FlyBase [53] were used for analyses of Gene Ontology (GO) (GOTERM_BP_DIRECT category), publication enrichment, and gene function analysis. The expression patterns of some gene groups that were listed from previous data were also examined. The list of infection-responsive core genes, housekeeping genes, and Ada2b-regulated (-independent) genes was obtained from previous studies [22,30,54]. Especially to obtain the list of Ada2b-regulated genes, we chose the genes upregulated in the *Ada2b* mutant from part A of Table 2 in [30]. We also utilized part B of the same table to obtain the list of Ada2b-independent genes. Data of the genes showing extremely low level of expression (average read counts < 10 on the row data of RNA-Seq) was eliminated for heatmap (fold-change) analyses.

### RT-PCR analysis

The expression levels of *Ada2b*, *RpL32*, *Drosomycin*, *CG6675*, and *CG33462* were determined using RT-PCR. To prepare fly samples, Ml or Sa suspension (OD = 1) was injected into flies as described above. The flies were collected at 4, 8, and 16 h post-challenge infection. Total RNA was extracted from three flies for each sample, and three samples were analyzed as biological replicates for each experimental condition. Complementary DNA (cDNA) was synthesized from total RNAs using ReverTra Ace (Toyobo), following the manufacturer’s instructions. RT-PCR analysis was performed using the Thunderbird Next SYBR qPCR Mix kit (Toyobo) or FastStart DNA Master SYBR Green I (Roche) with Light Cycler 96 (Roche). The primers used for RT-PCR analysis are listed in S6 Table. The relative gene expression was quantified using the R program. The expression data were statistically compared using ANOVA, followed by Tukey’s HSD post-hoc test with the R program.

## Supporting information

S1 Fig Specificity of training effects. (A-F) Survival curves of the flies with various combinations of bacteria (representing training + challenge bacteria): Ml + Pa (A), St + Sa (B), Ss + Pa (C), Ss + Sa (D), Ec + Pa (E), and Ec + Sa (F). Sa (OD = 0.2) was used in these experiments. Asterisks and NS indicate statistically significant (p-value < 0.05) and not significant, respectively, in Log-rank test. Numbers of flies used in these experiments are (A) 57, 56 flies / 3 vials, (B) 128, 135 flies / 6 vials, (C) 50, 56 flies / 3 vials, (D) 103, 117 flies / 6 vials, (E) 154, 158 flies / 9 vials, and (F) 57, 52 flies / 3 vials (with or without training, respectively).
(EPS)

S2 Fig Endurance of Ml-training effects. (A) Survival curves of the flies with Ml-training and Sa-challenge with 12-day interval. Survival rates under control and training conditions in this graph were measured from independent experiments. (B) Survival curves of the flies with Ml-training and Sa-challenge with 23-day interval. Asterisks and NS indicate statistically significant (p-value < 0.05) and not significant, respectively, in Log-rank test. Numbers of flies used in these experiments are (A) 54 flies / 3 vials, 75 flies / 4 vials, and (B) 38, 40 flies / 3 vials (with or without training, respectively).
(EPS)

S3 Fig Longevity of trained flies. (A) Long-term survival curves of the flies with Ml-training. (B) Long-term survival curves of the flies with St-training. Asterisks and NS indicate statistically significant (p-value < 0.05) and not significant, respectively, judged by Log-rank test. Numbers of flies used in these experiments are (A) 87, 95 flies / 5 vials and (B) 97, 95 flies / 5 vials (with or without training, respectively).
(EPS)

S4 Fig Expression patterns of DEG groups of MA system. (A-F) Box plots of z-scores of DEG groups for MA2 (A), MA4 (B), MA5 (C), MA6 (D), MA7 (E), and MA8 (F).
(EPS)

S5 Fig Expression patterns of DEG groups of TP system. (A-I) Box plots of z-scores of DEG groups for TP2 (A), TP3 (B), TP4 (C), TP5 (D), TP6 (E), TP7 (F), TP8 (G), TP9 (H), and TP10 (I).
(EPS)

S6 Fig Expression patterns of AMP genes. (A, B) Expression of *Drosomycin* gene in MA (A) and TP (B) systems. (C, D) Expression of *CecropinA1* gene in MA (C) and TP (D) systems. (E, F) Expression of *Attacin-A* gene in MA (E) and TP (F) systems. Expression levels are indicated as the normalized cpm (counts per million reads). Different alphabet letters indicate statistically significant differences (ANOVA, Tukey HSD post hoc test, p-value < 0.05).
(EPS)

S7 Fig Expression patterns of the infection-responsive core genes. (A, B) Heat maps of log2 fold-changes from mean levels of expression for the infection-responsive core up-regulated genes (163 genes; see Materials and methods) in MA (A) and TP (B) systems. (C, D) Heat maps for the infection-responsive core down-regulated genes (82 genes) in MA (C) and TP (D) systems. (E, F) Heat maps for the house keeping genes (30 genes; see Materials and methods) in MA (E) and TP (F) systems. The genes were sorted by the order of expression levels under training plus challenge conditions, and were listed on the right side of the heatmaps.
(EPS)

S8 Fig GO analysis for clustering groups in MA system. GO analysis (GOTERM_BP_DIRECT category) for each clustering group was performed. Two representative GO terms are shown here. Bar graphs show p-values of each GO term, as -log10 values. Numbers under the top of bars indicate numbers of genes matched to the GO terms.
(EPS)

S9 Fig GO analysis for clustering groups in TP system. GO analysis (GOTERM_BP_DIRECT category) for each clustering group was performed. Two representative GO terms are shown here. Bar graphs show p-values of each GO term, as -log10 values. Numbers under the top of bars indicate numbers of genes matched to the GO terms.
(EPS)

S10 Fig Expression patterns of the Ada2b-regulated genes. (A, B) Heat maps of log2 fold-changes from mean levels of expression for the Ada2b-regulated immune-related genes (A; 62 genes; see Materials and methods) and the Ada2b-independent immune-related genes (B; 30 genes) in MA system. (C, D) Box plots of log2 fold-changes from mean levels of expression for the Ada2b-regulated immune-related genes (C) and the Ada2b-independent immune-related genes (D). Asterisks and NS indicate statistically significant (p-value < 0.05) and not significant, respectively, in Kruskal-Wallis ANOVA and post-hoc Wilcoxon rank sum test.
(EPS)

S11 Fig Expression of *Ada2b* and its putative targets. (A) RT-PCR analysis measured relative expression of the *Ada2b* gene (against the *RpL32* control gene). The *Ada2b RNAi* line (*da > Ada2b RNAi*) significantly reduced expression of *Ada2b* in comparison with the control line (*da > GFP RNAi*). Asterisks indicate statistically significant (p-value < 0.05) in Student t-test. (B, C, D) RNA-Seq data for *Ada2b* (B), *CG33462* (C), and *CG6675* (D) genes during Ml-training and Sa-challenge conditions in MA system. Expression levels are indicated as the normalized cpm (counts per million reads). Different alphabet letters indicate statistically significant differences (ANOVA, Tukey HSD post hoc test, p-value < 0.05).
(EPS)

S12 Fig Knockdown of *Ada2b* in hemocytes. Survival rates of the flies with prior training of Ml bacteria (OD = 1; training) or saline (control) and subsequent Sa-GFP challenge (OD = 0.1). Asterisks and NS indicate statistically significant (p-value < 0.05) and not significant in Log-rank test, respectively. The genotypes of tested flies were (A) *hmlΔ > GFP RNAi*, and (B) *hmlΔ > Ada2b RNAi*. Numbers of flies used in these experiments are (A) 48, 56 flies / 3 vials, (B) 118, 110 flies / 6 vials (with or without training, respectively).
(EPS)

S1 Table Summary of RNA-Seq analysis. 21 RNA samples were sequenced by Illumina HiSeq 2000. Primary read data (36-base length) were deposited in DDBJ (DRA008187). After removing of adaptor sequences, the cleaned reads were mapped on the *Drosophila melanogaster* reference genome (ver. 6.04). Although most of them were primarily or secondarily mapped to a genomic region, some were unmapped to any region. Mapping rates are similar among samples.
(XLSX)

S2 Table Numbers of DEGs detected from pair-wise comparisons. DEGs was detected from pair-wise comparisons using a criterion (FDR-adjusted p-value < 0.05). Some DEGs were up- or down-regulated in the Y condition compared to the X condition. Some DEGs were up- or down-regulated more than 2-fold. Numbers of DEGs were indicated for each comparison.
(XLSX)

S3 Table List of DEGs from pairwise comparisons. Flybase IDs of the DEGs detected from pairwise comparisons of experimental conditions (e.g. conditionA_vs_conditionB).
(XLSX)

S4 Table GO analysis for MA clustering group. Top 15 (or less) of the GO terms (GOTERM_BP_DIRECT category) enriched to each clustering group of MA system were listed. Numbers of the genes belonging to the GO terms, rates among the genes of each clustering group, and p-values of enrichments were indicated.
(XLSX)

S5 Table GO analysis for TP clustering group. Top 15 (or less) of the GO terms (GOTERM_BP_DIRECT category) enriched to each clustering group of TP system were listed. Numbers of the genes belonging to the GO terms, rates among the genes of each clustering group, and p-values of enrichments were indicated.
(XLSX)

S6 Table Primers used in this study. Sequences of primers used in this study are listed.
(XLSX)

S1 Data Primary data of transcriptome in MA system. Normalized read counts of each gene were indicated for all samples of MA systems. Name of sample was showed in S1 Table.
(ZIP)

S2 Data Primary data of transcriptome in TP system. Normalized read counts of each gene were indicated for all samples of TP systems. Name of sample was showed in S1 Table.
(ZIP)

S3 Data Z-scores of DEGs categorized to the clustering groups in MA system. Z-scores from comparison between conditions (SS, MS, SA, MA in S1 Table) were indicated for 2077 DEGs. DEGs were divided into 8 clustering groups (name).
(ZIP)

S4 Data Z-scores of DEGs categorized to the clustering groups in TP system. Z-scores from comparison between conditions (SS, TS, SP, TP in S1 Table) were indicated for 5965 DEGs. DEGs were divided into 11 clustering groups (name).
(ZIP)
